# EXPOSURE TO NATURE IS RELATED TO GENERATIVITY, ESPECIALLY AMONG ADULTS WITH LIMITED FUTURE TIME PERSPECTIVE

**DOI:** 10.1093/geroni/igad104.0500

**Published:** 2023-12-21

**Authors:** Jana Nikitin, Fiona Rupprecht

**Affiliations:** University of Vienna, Vienna, Wien, Austria; University of Vienna, Vienna, Wien, Austria

## Abstract

Theory and research suggest that connection with nature may promote prosocial and environmentally sustainable behaviors. The first objective of the present study was to replicate this finding in an age-heterogeneous sample of N = 115 participants (Mage = 38, SDage = 17, age range 18-85) who reported their exposure to nature up to eight times a day for seven days. We examined the relationship between nature exposure and participants’ generativity, i.e., the extent to which they care about future generations and are politically, socially, and environmentally engaged. The second goal of the study was to show that this relationship is particularly pronounced among individuals with a shorter future time perspective that is related to remaining lifetime and associated with the choice of more emotionally significant goals that focus on generativity. We therefore hypothesized that nature experiences would be more strongly associated with generativity among adults whose future time perspective is limited. We found that people who were more exposed to nature in their daily lives also reported higher levels of generativity. This relationship was moderated by future time perspective. As hypothesized, the association was stronger among adults with a limited future time perspective. Contrary to the widely held stereotype that it is primarily younger adults who are concerned about climate and the future of humanity, we showed the opposite: People who perceived their remaining lifetime as limited were the ones for whom nature experiences were more strongly associated with concern for the environment and future generations.

